# Qualitative Dynamics of Suspended Particulate Matter in the Changjiang Estuary from Geostationary Ocean Color Images: An Empirical, Regional Modeling Approach

**DOI:** 10.3390/s18124186

**Published:** 2018-11-29

**Authors:** Dinghui Shang, Huiping Xu

**Affiliations:** 1 School of Ocean and Earth Science, Tongji University, Shanghai 200092, China; 1410845@tongji.edu.cn; 2 Institute of Deep-sea and Engineering, Chinese Academy of Science, Sanya 572000, China

**Keywords:** suspended particulate matter, the Changjiang Estuary, GOCI, spatial-temporal variability

## Abstract

The suspended particulate matter (SPM) in Changjiang Estuary is characterized by a high concentration of significant diurnal dynamics. With a higher temporal resolution (eight images obtained per day), Geostationary Ocean Color Imager (GOCI) was selected as the primary remote sensor for the dynamics monitoring in this paper, instead of other satellite sensor working in polar orbit. Based on the characteristics of the field spectra measured in the estuary, an empirical model was established with the band ratio of R_rs_745 divided by R_rs_490 and proven effective in Suspended Particulate Matter (SPM) estimation (R^2^ = 0.9376, RMSE = 89.32 mg/L). While, Validation results showed that the model performed better in coastal turbid waters than offshore clear waters with higher chlorophyll-a concentration, stressing the importance of partitioning SPM into its major components and doing separate analysis. The hourly observations from GOCI showed that the diurnal variation magnitudes exhibited clear regional characteristics, with a maximum in the turbidity belt near the mouth and a minimum in the offshore deeper areas. In addition, comparing the monthly averaged SPM distribution with the amount of sediment discharged into the estuary, the variation in estuarine turbidity maximum zone is more likely contributed by the sediments resuspended from the sea bed that has already accumulated in the estuarine delta.

## 1. Introduction

Suspended particulate matter (SPM) is a dominant constituent of coastal case-2 water, and it is an important parameter for coastal aquatic ecosystem by increasing water turbidity, reducing the euphotic zone depth and thereafter limiting ocean primary production [1,2]. In offshore waters, suspended particles are mostly organic (POM): phytoplankton and biomass debris, but inorganic materials (PIM) such as silt, clay and sand, are the dominant component in inshore waters [3,4]. Moreover, sediment particles are good carriers for nutrients and pollutants, so SPM plays a key role in coastal biogeochemical processes [5]. Researches on SPM distribution characteristics in the estuary areas are conducive to estimating the impacts of terrestrial inputs and human activities on the offshore water environment. Before satellite sensors were used in ocean environment monitoring, properties of seawater were usually determined by samples collected during cruises, which cost much more time and money [6,7]. In contrast, satellite sensors can take down the biological optical properties of seawater at a high temporal and spatial resolution, from which we can obtain the variation features of SPM concentration [8,9,10,11]. Lahet and Stramski [12] proposed to use the optical properties acquired from MODIS images to extract the turbid plume from surrounding water masses in San Diego coastal waters. In the case of Congo plume, Hopkins et al. [13] used four satellite-derived parameters: temperature, salinity, chlorophyll, and sea level to ascertain its dispersal pattern. Except for Moderate-Resolution Imaging Spectroradiometer (MODIS) [4,10,11], some other sensors installed on the polar-orbit satellites, such as Medium Resolution Imaging Spectrometer (MERIS) [14,15], and Visible and Infrared Imager/Radiometer (VIIRS) [7,16], have been used to map SPM dynamics in various regions in recent decades. However, they can only get one image per day at mid-low latitudes separately, far from meeting the requirements on short-term dynamics observation, which is critical for understanding the drastic change associated short-term events. The Geostationary Ocean Color Imager (GOCI) launched on 27 June 2010 by South Korea as the first synchronous satellite, is uniquely capable of monitoring short term and regional oceanic phenomena (e.g., tide dynamics, red tides, river plumes, and sediment transport) with a high spatial resolution (500 m) and a very high temporal resolution (1 h, eight images per day) [17], which makes it very useful for monitoring the diurnal dynamics of the material in coastal areas.

A lot of models have been established to estimate SPM concentration by remotely sensed data, including empirical [4,10,11,12,13,14,15,18,19,20,21,22,23,24] and semianalytical models [16,25,26]. However, semianalytical models are usually based on optical theories to carry out rigorous deduction with accurate inherent optical properties (IOPs), which cannot be measured accurately [27]. Empirical algorithms are based on the linear or non-linear relationship between water optical properties in specific bands and SPM concentration. These algorithms are normally derived from in situ measurements with undefined single SPM unity, which can be highly variable in natural environments, and thus are limited by regional applicability [28]. Limited by the field measurements and alternative bands, we just listed several algorithms that can expand to GOCI data with appropriate bands (Table 1).

Changjiang Estuary is known with massive sediment discharge from Yangtze River, and affected by resuspension of bottom sediments and anthropogenic activities such as agriculture and sewage discharge, contributing to heterogeneous optical properties. Thus, a refined model is needed to obtain accurate values of SPM concentration from satellite data. In this study, we established an empirical relationship between R_rs_ and SPM concentration collected during four cruises, which can be expanded into GOCI data for dynamics monitoring. For the complex optical property in coastal waters, the other objective was to find a proper atmospheric correction algorithm for GOCI images. Based on the proper atmospheric correction algorithm and the applicable model for the study area, we try to extract diurnal variability and long-term trend of SPM distribution, and to analyze the main forces for the variation with other ancillary data. 

## 2. Dataset and Methods

### 2.1. Study Area

**Figure 1:**
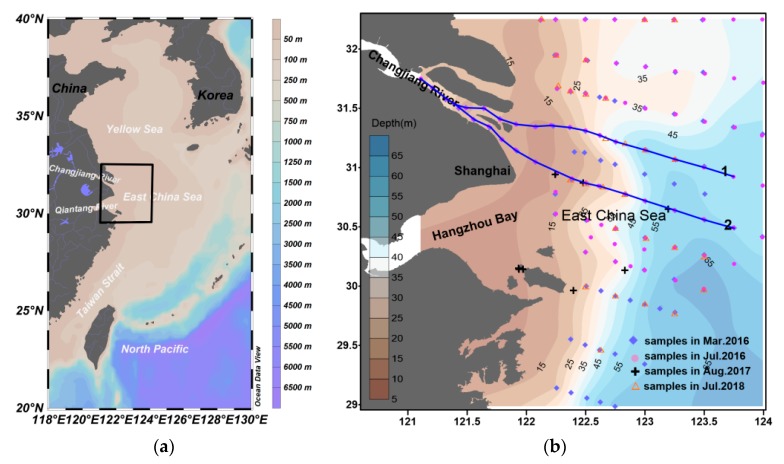
(**a**) Location of the study area; (**b**) Detailed distribution of the samples during four cruises. Blue lines represent two transects selected for further analysis in later sections, transect 1 including section B and A4, and transect 2 including section C and A6.

The sample area is mainly concentrated in the Yangtze River estuary, Hangzhou Bay and its adjacent sea areas, locating at western of the East China Sea (ECS) (Figure 1). The ECS is one of the three marginal seas in China, with approximately 486 million tons of sediments input from the Yangtze River, Qiantang River, and other small rivers annually. The Yangtze River Sediment Bulletins showed that the average annual runoff and sediment discharge from the Yangtze River reach 9.04 × 10^11^ m^3^ and 1.307 × 10^8^ ton between 2010 and 2016. Comparing to the data survived by Milliman and Syvitski in 1992 [29], the mass of 4.8 × 10^8^ tons sediment was decreased largely in the last two decades due to the Three Gorge Dam [30,31], among which 2~5 × 10^6^ tons were organic matter [32]. In addition, there is about a volume of 42 km^3^ diluted water was poured into the ECS and the annual sediment discharge can reach 7.9 × 10^6^ ton together with other small branches [33]. Such large quantities of river water, terrestrial sediments, and associated chemical species like nutrients and pollutants delivered into the Changjiang Estuary have substantial impacts on the biogeochemistry cycle of this region. 

### 2.2. Introduction of Dataset

Four types of data were used in this study. These included GOCI daily data in 2016, in-situ remote sensing reflectance (R_rs_) and SPM measurements, surface runoff and sediment discharge observations from Datong hydrographic station, and remotely sensed winds.

#### 2.2.1. Field Spectral and SPM Collection 

Four cruise surveys were conducted in March 2016, July 2016, August 2017 and July 2018 in the ECS, from which the SPM concentration, and R_rs_ were collected (Table 2). According to the ocean color protocol [34], Hyper-spectral reflectance of water surface was recorded by different instruments in the four cruises. During the March 2016 cruise spectral measurements were carried out by the Satlantic, Inc.’s Hyperspectral Surface Acquisition System (HyperSAS) operating in 349–858 nm. The radiance sensor has two optic probes so that it can be pointed to the sea and sky to acquire spectrum at the same time. The nadir and zenith angles were kept the same, generally between 30°and 50°, which can effectively avoid the shadow of the ship on the water surface and the influence of direct solar radiation. And the other three cruises in July 2016, August 2017 and July 2018 were collected by an ASD Fieldspec HandHeld 2 with a spectral range from 325 to 1075 nm. Every station we collected radiance of water surface and a standard reflecting plate as well as skylight in three times with the zenith and azimuth angles at 40°and 135°. As integration time of ASD was chosen according to the intensity of radiance received by the detector, so we should do optimization before local measurement. Fifteen curves for each target and two duplicates for each station were recorded. Then the water remote sensing reflectance (R_rs_) was derived from in situ measurement as:

(1) $$R_{\mathrm{rs}}=\frac{{[L}_{t}{(\lambda )-\mathrm{rL}}_{\mathrm{sky}}{]\times \rho }_{P}}{{\pi \times L}_{P}}$$

where L_t_, L_sky_, L_P_ represents the radiance signal values captured for water surface, skylight and standard plaque separately. r refers to the air–sea interface reflectance, depending on sky radiance, solar zenith angle, wind speed and the instrument’s viewing angle. Based on Mobley’s earlier research [35] and the measuring environment during cruises, r was set to 0.025 for homogeneous sky conditions and less than 5 m/s wind speed. $\rho_{P}$ stands for the reflectance of the standard plate, which was calibrated to 99.99% before the cruises. The turbidity of the study area was quite high so that the bottom scattering in the measurement of water surface reflectance could be neglected.

Simultaneously with the filed water leaving reflectance measurements, sufficient samples of surface water (depth fixed at about 1.5 m) were collected to estimate the concentration of suspended particle material gravimetrically. The method was outlined in Strick and Parsons (1972) [36]. For the convenience of storing, all samples were filtered on boat with pre-weighed 0.45-μm cellulose acetate filters or Whatman GF/F filters. It should be mentioned that the salinity of sea water is always high and all filtered filters need to be rinsed with 20 mL distilled deionized water three times before placing in plastic petri dishes. All filters were frozen and stored in below −20 ℃ freezer until laboratory procedures. The filters were dried in an oven at 75 ℃ for about 2–3 days and then placed in a drier for 4–5 h to cool to the room temperature. The dry weights of the filters were measured repeatedly with an electronic analytic scale until the difference of two successive SPM concentration measured was within 0.01 mg/L. Table 2 shows statistical descriptions of all the in situ data collected in four cruises. 

#### 2.2.2. GOCI Data

GOCI is the first ocean color sensor on board a geostationary satellite with a spatial resolution of about 500 m. It acquires data in eight spectral bands (412 nm, 443 nm, 490 nm, 555 nm, 660 nm, 680 nm, 745 nm and 865 nm). Compared to other sensors, its outstanding advantage is that it has a revisit time of around 1 h and can get eight images per day. Its higher temporal resolution greatly improves the condition that coastal areas always are contaminated by clouds. The GOCI The GOCI observing area is about 2500 km × 2500 km (116.08° E–143.92° E, 24.75° N–47.25° N for central directions) centered on 130° E and 36° N, covering the coasts of Eastern China, the Korean peninsula, and Japan, along with the corresponding shelves and open oceans [22].

About three hundred of GOCI level-2C Rayleigh corrected reflectance data were acquired from the Korea Ocean satellite center (KOSC: http://kosc.kiost.ac.kr/eng/) throughout 2016, including nine days (1, 15, 16, 19 March and 4, 5, 6, 7 15 July 2016) corresponding to our cruises date. As the data was already Rayleigh scattering corrected, we just need to select the appropriate aerosol inversion model to remove the effects of aerosol scattering, which will be presented in Section 2.3. 

#### 2.2.3. Ancillary Data

Monthly Blended Sea Winds data in 2016 were downloaded from the remote sensing systems (http://www.remss.com/measurements/ccmp/). The cross-calibrated multi-platform (CCMP) gridded surface vector winds are produced using satellite, moored buoy, and model wind data, and as such, are considered to be a Level-3 ocean vector wind analysis product [37]. These data have a spatial resolution of 0.25° × 0.25° (~27.5 km at the equator) and a temporal resolution of 6 h. The data covering the study region were extracted and resampled to match the GOCI resolution (500 m).

Surface runoff and sediment flux data were collected at Datong station (the hydrological station of the Yangtze River closest to the river mouth), representing the total materials discharged from the Yangtze River to the estuary. Monthly hydrological data in 2016 were obtained from the Changjiang Sediment Bulletins (http://www.cjh.com.cn).

Previous research has shown that the concentration of ozone affects its absorption capacity at visible bands, especially in the green. For the purpose of removing the effect of atmosphere exactly, daily ozone concentration data corresponding to the GOCI images derived from TOMS-like instrument was also collected from NASA Ocean Color website in this study. 

### 2.3. Aerosol Scattering Correction 

At satellite altitude, apart from the radiance contributed by the atmospheric molecules Rayleigh scattering, aerosol scattering, sea surface reflectance, the water-leaving radiance received by the satellite sensors is only about 5–10 percent [38]. A statistical study showed that researchers should keep the accuracy around 0.1% on atmosphere correction to process nLw within 5% [39] of error. So atmosphere correction becomes a key process for quantitative analysis on satellite ocean color productions. At present, the atmospheric correction algorithm for open ocean clean waters has been widely validated and recognized, like currently implemented by the NASA Ocean Biology Processing Group (OBPG) for derive ocean color data from SeaWiFS, MODIS and VIIRS sensors. However, the turbid coastal areas, featured by significant spatial and temporal variation, are difficult to extract spectral characteristics. 

**Figure 2:**
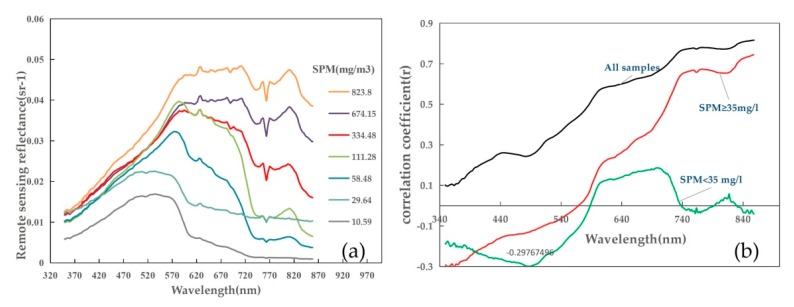
(**a**) The field measured spectral with different SPM concentrations in the study area. (**b**) The correlation coefficient (r) between the field measured SPM concentration and R_rs_ varied with the wavelength. In the figure, the black, red and green line were calculated respectively based on the whole (N = 48, 5.51–2061.3), high (N = 26, SPM ≥ 35 mg/L) and low (N = 22, SPM < 35) SPM concentration ranges.

The developed atmospheric correction algorithms for costal turbid areas are mostly based on the assumption that the reflectance of water in the short-wave infrared (SWIR) region is expected to close to zero, which is also valid in turbid waters [40]. However, there are no SWIR bands configured on the GOCI, whose maximum band is 865 nm. So here two algorithms with no SWIR bands used for aerosol scattering correction for turbid waters have been presented and compared, to find an appropriate algorithm for the study area. One is the standard algorithm integrated in the GOCI Data Processing System (GDPS:version 1.4). This algorithm is constructed by assuming that the strong relationship between satellite-derived ocean reflectance at red and NIR bands in the turbid coastal waters is dominantly varied with the concentration of inorganic suspended particulates [41]. Another is proposed by He [42] to apply in SeaWiFS imagery for coastal turbid waters with the assumption that the water-leaving radiance at ultraviolet wavelengths could be neglected compared with longer wavelengths. As the in situ measured spectrum show in Figure 2, the remote sensing reflectance at 865 nm band will surpass its results at 412 nm band when the concentration of SPM is larger than 100 mg/L. The performance of this algorithm has been validated in current ocean color sensors [42] and other costal turbid waters [43], and the details of the application in GOCI images can be found in his latter researches [18,42]. As GOCI doesn’t have ultraviolet either, we use 412 nm band to be a substitution. The comparative analysis of the R_rs_ data derived from the two methods showed that the UV-AC performed better in our study area. All GOCI-level 2C data were subsequently processed with the UV-AC algorithm. It’s worth noticing that solar zenith angles used in this study were obtained base on the general solar position calculation algorithms contributed by NOAA. Comparing with generating solar geometries from the level-1B files [19]; it could significantly reduce time cost and simplify the processing procedure.

### 2.4. GOCI Processing 

About 288 GOCI images were acquired during the year 2016 to analysis the variation of SPM concentration distribution characteristics in the Changjiang Estuary. All images were processed in the following steps:

(1) Images with less cloud covering were selected by the browsing images included in every image zip file.
(2) Using GDPS batch processing tool, all images were converted a to the Environment for Visualizing Images (ENVI) format to carry out following steps with some scripts in IDL (interface description language), which specializes in satellite data processing.
(3) Making a binary mask to remove land areas and patches contaminated by clouds. The land mask keeps the same for the fixed scan area of GOCI and can be downloaded on the website, but the cloud cover was always different from each other. Here it was derived from its unsupervised classification results.
(4) Using the proper atmospheric correction method to obtain satellite-derived R_rs_ from GOCI images. It’s worth noticing that solar zenith angles used in computing the transmissivity were obtained base on the general solar position calculation algorithms contributed by NOAA.
(5) Several scripts developed in IDL were used to combine area, mask, re-project, clip, correct aerosol scattering, and calculate SPM concentration orderly.
(6) Mapping SPM concentration from GOCI in our study area in ArcGIS.

In addition, to get matching pairs for subsequent validation, in situ data collected during the three cruises were used to match with the GOCI images in the seven bands (412, 443, 490, 555, 660, 680, 745 nm). The original level 1B data used in the study needs atmosphere correction to derive remote sensing reflectance. Because of the heavy cloud cover and frequent variation in the study area, we set two criteria: (1) matched with cloud-free images with a time interval less than 1 h; (2) satellite remote sensing reflectance were extracted from a window size of 3 × 3 pixels centered at the location of the in situ sample, and only the valid pixels in the window is more than 4 can be counted. As in-situ spectral samples can only be measured during 9:00 a.m.–15:00 p.m. and clear skies, the in-situ spectral samples is far fewer than SPM samples, and there are 14 spectral samples and 28 SPM samples were picked out of the all 173 samples.

### 2.5. Application of the GOCI Spectral Response Function for Field Spectral

The in-situ spectral samples need to be convoluted with the GOCI spectral response functions to get the equivalent reflectance at the central wavelengths of GOCI using Equation (2) prior to further analysis in model development and validation [44]:

(2) $$R_{\mathrm{rs}}\left(\lambda_{n}\right)\;=\;\frac{\int_{\lambda_{i}}^{\lambda_{j}}F_{0}{(\lambda )S}_{n}{(\lambda )R}_{\mathrm{rs}}(\lambda )d\lambda }{\int_{\lambda_{i}}^{\lambda_{j}}S_{n}{(\lambda )F}_{0}(\lambda )d\lambda }$$

where λ_i_ and λ_j_ represents the start and end wavelengths for every band (λ_n_). F_0_(λ) is the solar irradiance. S_n_(λ) is the relative spectral response function at band n and it is always different with sensors. 

### 2.6. Statistical Criteria for Model Evaluation

The accuracy evaluation in the study were conducted by comparing satellite-derived and in situ measurements with three parameters including root mean square error (RMSE), the Correlation Coefficient (r) and the mean absolute relative error (MARE), which are computed as following equations:

(3) $$\mathrm{RMSE}\;=\sqrt{\frac{\sum_{i=1}^{n}{{(x}_{i}{-y}_{i})}^{2}}{n}}$$

(4) $$r\;=\frac{n\sum^{}x_{i}y_{i}-\sum^{}x_{i}\sum^{}y_{i}}{\sqrt{n\sum^{}x_{i}{}^{2}-{\left(\sum^{}x_{i}\right)}^{2}}\sqrt{n\sum^{}y_{i}{}^{2}-{\left(\sum^{}y_{i}\right)}^{2}}}$$

(5) $$\mathrm{MARE}\;=\frac{\sum_{i=1}^{n}{|(x}_{i}{-y}_{i}{)/y}_{i}|}{n}$$

where n refers to the number of samples, x_i_ is the satellite-derived result and y_i_ is the corresponding values measured in situ.

## 3. Results 

### 3.1. Spectrum Characteristic Analysis

Figure 2a shows field measured spectral reflectance curves for different SPM concentrations. Similar to other coastal case-2 waters [10,25], the R_rs_ is highly variable with different range of SPM concentration over the visible and near-infrared spectral regions in the study area. It is obvious that the magnitude of the reflectance increases with the increment of SPM concentration. And R_rs_ in different wavelength intervals shows respective characteristics. Obviously, R_rs_ in 350–490 nm increases slowly with wavelength and few changes appear with the variation of SPM concentration. In comparison, there are more spectral features found in the longer wavelength (>490 nm). First of all, the location of the break point in green region (490–570 nm) appears to move to longer wavelength with the increment of SPM concentration. Secondly, when the SPM concentration is larger than 100 g/m^3^, there is another peak in near-infrared range (~810 nm) whose magnitude increases distinctly with SPM concentration. Comparing with the sampling map, samples with SPM larger than 100 mg/L usually located in mineral dominated coastal waters, while samples below 100 mg/L mostly located in organic dominated offshore waters. So it is probably the result of a higher backscattering by mineral suspended particles than organic matters like phytoplankton and biomass detritus. Finally, in extremely turbid waters (SPM > 385 g/m^3^), and the reflectance spectrum presents a broad peak between 590 nm and 710 nm and the distinct peak at ~810 nm can reach the same magnitude. The broad peak was mainly contributed by the same magnitude of backscattering coefficient and absorption coefficient at red band in global [25]. It’s interesting that the spectrum with SPM ~29.64 mg/L showed the same downward trend in red and NIR regions (>620 nm), but the magnitude is nearly get to the level of SPM ~111.28 mg/L at 740–780 nm. The observation presents the complex inherent optical properties of the offshore waters (SPM < 35 mg/) with uncertain concentration of organic matter, phytoplankton and mineral mass to some degree.

The correlation coefficient (r) calculated by wavelength within the spectral range of GOCI (Figure 2b) appears that the reflectance at longer bands (red and NIR) is more sensitive to SPM concentration (black line). For instance, the linear correlation coefficient r increases from 0.249 at 490 nm to 0.771 at 745 nm. This observation is mostly caused by the higher contribution of backscattering coefficient on the reflectance with the increment of wavelength. However, there is no obvious correspondence in slightly turbid waters, with a best result at ~490 nm (r = −0.3). The negative correlations in Figure 2b below about 550 nm in the two groups (SPM above or below 35 mg/L) indicate increasing mineral affect and decreasing organic effect in the organic dominated shorter wavelengths.

### 3.2. Regional Tuning Model for GOCI in the Changjiang Estuary 

There have been many models developed to estimate SPM concentration including empirical, semianalytical and physical with the development of satellite sensors, among which some were listed in Table 1. Except for the empirical models, a semianalytical model developed for Changjiang river estuary by Chenjun et al. [26] was also calibrated to estimate its accuracy. These algorithms can be divided into four categories, empirical model with single band (EM-SB), and empirical model with band ratios (EM-BR), empirical model with multi-bands (EM-MB), and semianalytical model (SAM). It should be noted that areas with different SPM composition (organic dominated or mineral dominated), particle size distribution, density and sensors, usually have particular sensitive bands and formula. As a result, the primary goal of the study is to establish an appropriate model on the basis of the field measured datasets that can be expanded to GOCI satellite data to estimate SPM concentration in the Changjiang Estuary.

The field dataset collected during first three cruises were sorted by SPM concentration, and one sample would be picked out every five samples to validate model accuracy. Then, we got 48 and 12 samples separately to compose the calibration dataset and the first validation dataset. The dataset acquired in July 2018 was used to further validate the performance of the optimal model.

**Figure 3:**
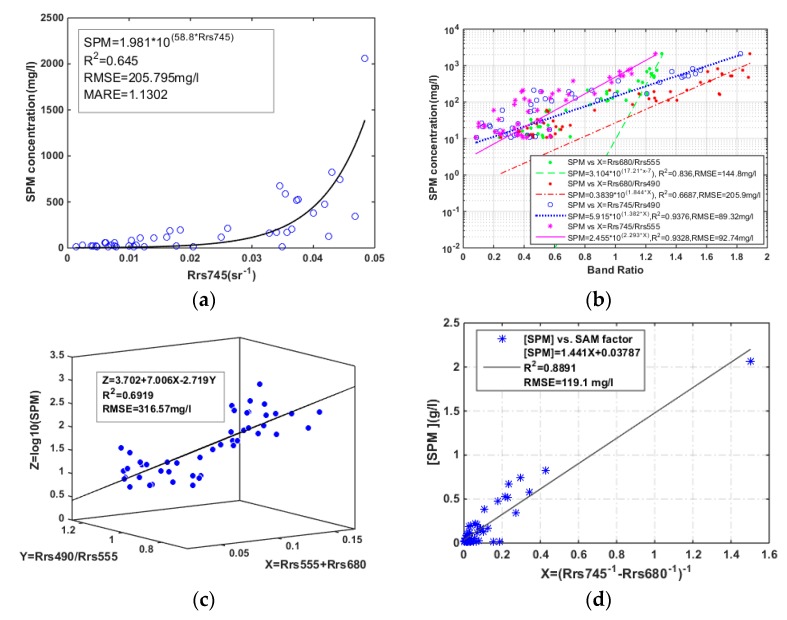
The relationship between the field measured SPM concentration and R_rs_-derived variables. (**a**) SPM concentration vs. R_rs_ at 745 nm. (**b**) SPM concentration vs. band ratios where λ_1_ at 745 nm or 680 nm, λ_2_ at 490 nm or 555 nm. (**c**) SPM concentration vs. combined R_rs_ at 490 nm, 555 nm and 680 nm. (**d**) SPM concentration (g/L) vs. the variable used in the SAM model.

Given the strong correlation between reflectance at the NIR band and SPM concentration, as shown in Figure 2b, the EM-SB model created in this paper is using the NIR band (745 nm), and explaining 65.4% of the SPM variation (Figure 3a). Nevertheless, samples with extremely high SPM concentration spread out around the fitting curve, contributing to the high root mean square error (RMSE) and the mean absolute relative error (MARE) (RMSE = 205.795 mg/L, MARE = 113.02%). The poor performance for high SPM concentrations and over estimation for low SPM concentrations indicate that single band cannot describe the relationship between reflectance and SPM concentration exactly. 

**Figure 4:**
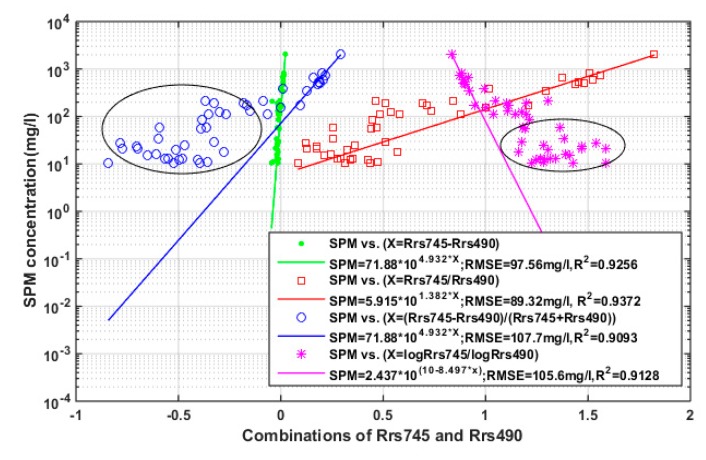
Comparisons of the models constructed on the basis of SPM concentrations (mg/L) and the combinations of reflectance at 745 nm and 490 nm, in the forms of difference of the two bands, SI and band ratio, or band ratio in log scale, respectively.

Shown as Figure 2b, samples with low and high SPM concentration have two different sensitive bands, 490 nm and 745 nm. We applied the two bands to develop an optimal algorithm for the study area. The variables used in the algorithms were basically classified into four combinations: difference of the two bands (R_rs_4745 − R_rs_490), sediment index (R_rs_745 − R_rs_490)/(R_rs_745 + R_rs_490), band ratio (R_rs_745/R_rs_490), and log ratio (log (R_rs_745)/log (R_rs_490)). The results were presented in Figure 4 with the SPM concentrations presented in logarithmic scale to exhibit their capability in different SPM ranges. Although the R^2^ and RMSE of the four algorithms seemed good, samples with medium and low SPM concentration (the blue and purple marks in black ellipses) were misestimated and far away from several fitting curves, except for the algorithm generated as Equation (6). The model can explain 93.72% of the SPM variability, and give the lowest RMSE of 89.32 mg/L.

(6) $$\{\begin{array}{c} {\mathrm{SPM}\;=\;10}^{0.772+1.382X}\\ {X\;=\;R}_{\mathrm{rs}}{745/R}_{\mathrm{rs}}490 \end{array}$$

Models with red and green bands were also recalibrated and presented in Figure 3b, with a similar problem that serious underestimation for lower SPM concentration (SPM < 100 mg/L). In comparison, theses algorithms are more sensitive for highly turbid samples.

The EM-MB models proposed by Zhang et al. [11] and Siswanto et al. [24] were based on the feature that the green band (555 nm) is sensitive to the SPM variation in slightly and moderately turbid waters, while the red band is sensitive to those in highly and extremely turbid waters. The calibration result (seeing Figure 3c) showed that this model isn’t applicable over the whole SPM range, with the tendency that overestimation in moderately turbid waters and underestimation in extremely turbid waters. By contrast, the SAM model constructed by Chen et al. [26] performed fairly well in SPM estimation (R^2^ = 0.8891, RMSE = 119.1 mg/L). However, there are still limitations in slightly and moderately turbid waters (Figure 3d), explaining the high MARE value of about 261.15%. A higher MARE value of 472.18% in samples with lower SPM concentration verified the fact that the model was established for high SPM values ranging from 100 to 1000 mg/L originally.

### 3.3. Model Validation

**Figure 5:**
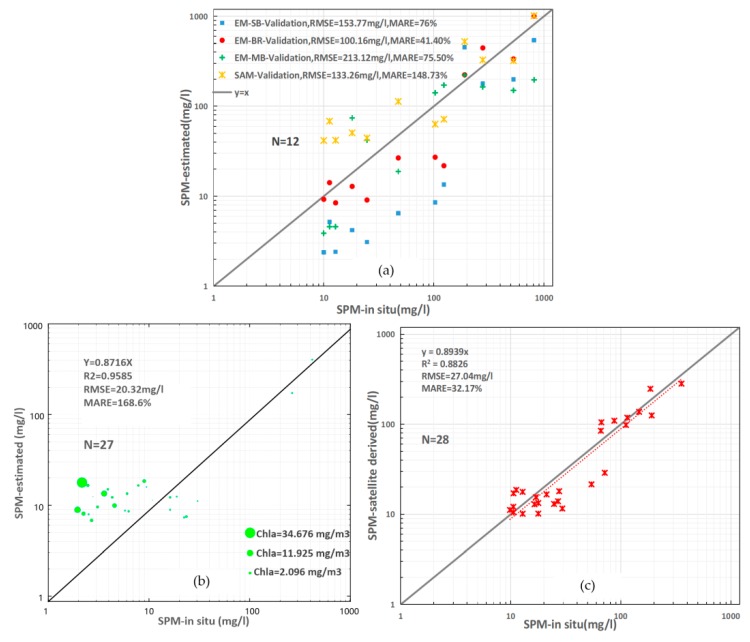
(**a**) Scatter plots of field measured vs. estimated SPM for the four kinds of algorithms with the field validation data. The EM-BR represents the retrieval model used in this manuscript. (**b**) Scatter plots of field measured vs estimated SPM with validation dataset measured during July 2018 cruise. The size represents chlorophyll-a concentration of each sample. (**c**) Validation results of applying the GOCI-derived Rrs in SPM retrieval model.

To further approve the applicability of the optimal algorithm used in this manuscript to estimate SPM concentration, we evaluated the performance using the two field validation datasets described in Table 2. The SPM concentration in the first validation dataset varied from 10 to 812.7 mg/L, felling into the range of calibration dataset. Significant differences were found among the comparisons of the measured and model-derived SPM concentrations (Figure 5a). The EM-MB model seems to be unsuitable for high SPM values, but adaptive for low SPM values with the maximum RMSE (213.12 mg/L) and a relatively lower MARE (75.5%). On the contrary, the SAM model gives the maximum MARE (148.73%) by overestimating in low SPM areas. The optimal model proposed in the study performed best with the minimum RMSE (100.16 mg/L) and MARE (41.4%).

The second validation dataset here was used to confirm the accuracy of the optimal model in areas with lower SPM concentration, whose components varies obviously with seasons. As presented in Figure 5b, samples in this dataset usually had a higher chlorophyll a concentration. It’s obvious that the model in slightly turbid waters cannot perform as well as in the turbid waters, especially with phytoplankton blooms. Although the overestimation in low SPM area is serious, the model is still promising in capturing the features of SPM diffusion after pouring into the sea from the estuary. 

We also used the pixels extracted from the GOCI images for the same geographical locations with in situ SPM sampling stations to validate the stability and accuracy of the model. Figure 5c shows the performance of the model in estimating SPM concentration in the Changjiang Estuary. We found that the model-derived SPM concentration has a good correlation with field measured SPM concentration, with the corresponding RMSE and MARE of 27.04 mg/L and 32.17%, respectively. 

### 3.4. Application to GOCI Images

#### 3.4.1. Atmospheric Correction

Although we have conducted three cruises to collect field data, only five days of GOCI images were chosen to validate the results of the two atmosphere correction algorithms mentioned above, with less cloud cover and matched the in-situ spectral samples. As the duration between satellite overpassing timing and measurement increases, the mismatch between the in situ and satellite derived SPM also increases depending on the spatial variability and water dynamics influenced by ocean currents and wind [25]. To account for the source of error of R_rs_ due to time difference between satellite image acquisition and in situ measurement, we calculated the average R_rs_ for a 3 × 3 pixels window. The 15 matching pairs within a 1-h time interval were picked out to compute the accuracy assessment factors mentioned in Section 2.6. The results in Table 3 show that the UV-AC algorithm presents a better performance in the study area with lower RMSE, MARE and higher correlation coefficient. And the UV-AC algorithm was used in later processing of GOCI images.

#### 3.4.2. Diurnal Variation of SPM Mapped by GOCI

**Figure 6:**
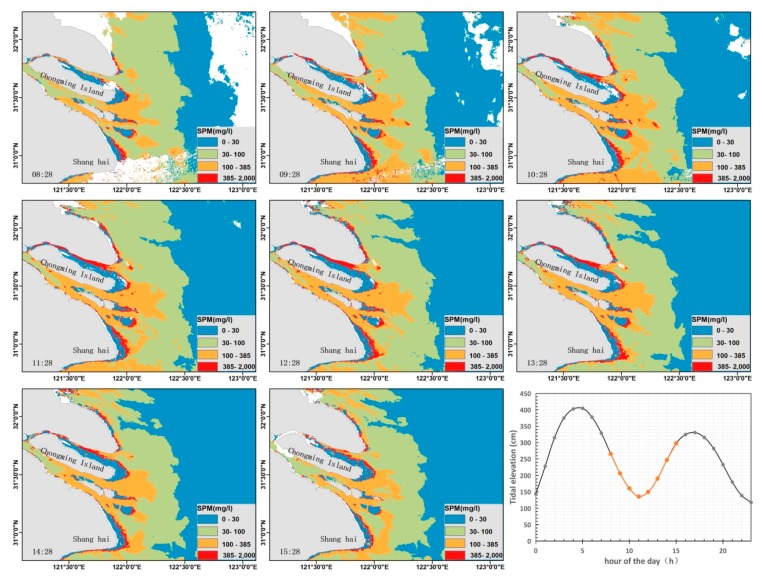
Hourly maps of SPM concentration in the Changjiang Estuary derived from GOCI on 16 March 2016. The labeled time in the image corresponds to the observation time in Beijing Time. The subplot at the lower right corner shows the hourly tide elevation from the tidal tables of the “Luchaogang” tide gauge station in the same day of the GOCI image.

Based on the satellite R_rs_ derived by atmospheric correction and the regional SPM retrieval model, we obtained eight hourly maps of the SPM concentration in the Changjiang Estuary, provided by less cloud contaminated GOCI data on 16 March 2016 (Figure 6). Here we divide the SPM concentration into four levels, which shows the turbid plume is predominately centered in front of the river mouth. When the diluted water flows through the delta, the SPM concentration has an apparent decline in all directions for the deposition with the decrease of the current speed and funnel topography (Figure 1b). 

Meanwhile, the second level of the SPM concentration (30 < SPM < 100 mg/L) almost spreads out through the shallow waters (depth < 40 m) and corresponds well with the topography. Given the hourly observation by GOCI, the diurnal variation of SPM concentration in the estuary is evident. The horizontal distribution of the moderate SPM concentration seems to be unchangeable during the eight hours, whereas the turbid plume varies in extent and magnitude. The magnitudes of diurnal variations in the estuary vary with geographic locations, and the further quantitative analysis results can be found in latter analysis. 

#### 3.4.3. Monthly Variation of SPM Mapped by GOCI

**Figure 7:**
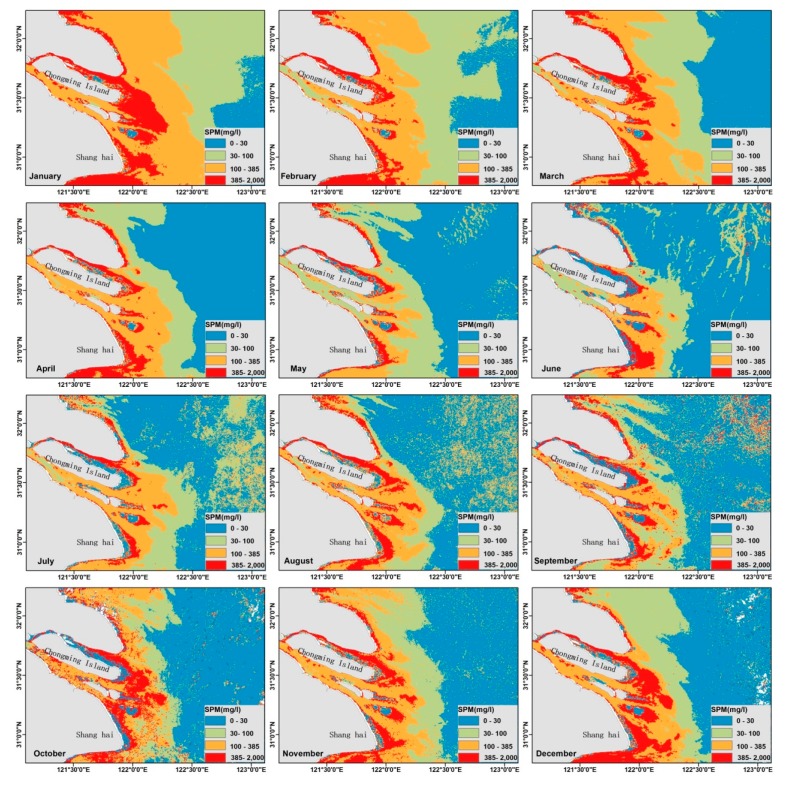
The GOCI-derived SPM distribution in the Changjiang Estuary during each climatological month in 2016.

The SPM retrieval model was applied to the 288 selected GOCI images throughout 2016, from which the monthly mean and monthly climatology SPM products were generated. Figure 7 shows the monthly SPM distribution in 2016. The highest SPM concentration locates near the mouth of the river. There are significant spatial gradients observed on the classification maps of each climatological month, as well as the clear seasonality. In general, the monthly maps of SPM concentration exhibit a similar trend in horizontal distribution, that the concentration decreases from >100 mg/L in nearshore waters to <30 mg/L in offshore waters.

The SPM concentration in the surface layer is high in winter (December, January and February) and spring (March, April and May), when the SPM contour of 30 mg/L can reach 35 m isobaths. Moreover, the highest value of the mean SPM concentration in the estuary is found in January, with maximum area of extremely turbid waters (SPM > 385 mg/L) around the delta, while the contour of 100 mg/L almost extends to 30 m isobaths. The axis of the turbid plume near the estuary extends southeastwards in winter. In contrast, the concentration of SPM is low in summer and the direction of the turbid plume deflects from southeast to northeast. The SPM contour of 30 mg/L is confined near the 25-m isobaths.

It’s interesting that the substantial sediment plume in the northern part of the study area is extending southeastward from Jiangsu coast to the Changjiang Estuary in winter, but vanishes in summer. The distribution of the sediment plume is consistent with previous studies [45,46]. As Dong et al. [47] reported, the dispersal of the sediment plume generated in the Yangtze Bank was restricted along the coast because of the frontal boundary between the Yellow Sea Cold Water Mass and the coastal water along the Jiangsu coast. According to the model results in Luo zhifa [48], the strong pycnocline caused by the thermocline and halocline in summer prevent the suspended sediment in the bottom diffusing to the surface layer. 

## 4. Discussion

### 4.1. Factors for SPM Retrieval Accuracy

The accuracy of SPM estimation in satellite data is usually affected by two factors: the performance of the retrieval model constructed with the field data, and atmospheric correction of the satellite images. The data quality is critical in model construction. Röttgers et al. [49] conducted research on quantifying individual measurement uncertainty in SPM concentration. The results showed that salt retention and loss of filter material during washing and drying procedures were common among most of the samples. This error varied with salinity and would be significant at lower concentrations of SPM [49,50]. As such, an experiment to calculate the error from salt retention in Changjiang Estuary is necessary in later researches.

The SPM concentration also plays an important role in retrieval accuracy. It is known that suspended materials contain organic and inorganic matters [3,4], whereas most research in coastal waters did not discriminate between the two. This was mainly due to the waters’ mineral-dominated nature, including the area focused in this research. However, the proportion of inorganic matter (mineral particle) decreases from shelf to offshore waters, contributing to a decline in backscattering. So it will touch the barrier when estimates SPM concentration in clean offshore waters, as the samples with low SPM concentrations scattered around the fitting curve irregularly (Figure 4 and Figure 5b). Moreover, the optical property is also affected by particle sizes in mineral-dominated waters [51]. Bower and Binding [3] found that the smaller sized inorganic sediments generally lead to a higher spectral reflectance. These problems will only be solved by a concerted effort at discriminating and measuring PIM and POM utilizing the recommendations of Stavn et al. [50] and Röttgers et al. [49]. Stavn and Richter [27] demonstrated the improvement in the northern Gulf of Mexico. Binding et al. [3] and Zhao et al. [4] conducted a successful estimation in mineral particle concentration. 

Since about 90% of the signal captured by the sensor is reflected by atmosphere [38], an atmospheric correction is also critical before using the model in processing satellite images. Here we compared two algorithms developed in turbid waters, and the results show that the algorithm based on the ultraviolet assumption is perform better. However, compared with the in-situ spectral samples (Figure 2a), the reflectance at 412 nm will surpass the value at red and NIR bands, which may cause overestimation on aerosol scattering reflectance. Furthermore, uncertainty from the other assumption of the algorithm that the aerosol scattering reflectance in the area is flat, should to be considered later. Gordon and Wang [52] reported that the flat spectrum approximation for the aerosol may cause large extrapolation errors at short wavelengths.

### 4.2. Driving Forces on SPM Variation 

For the diurnal variation, given the assumption that the fluvial discharge is unchanged during the day, tidal current is the main factor to be considered in the study. The Figure 6 displays SPM diurnal variation spatially only, from which we cannot distinguish the tidal-influenced area from perimeter zones. Here we analyzed the tidal force based on satellite images collected in nine days with less cloud contamination, almost one day per month in 2016. 

**Figure 8:**
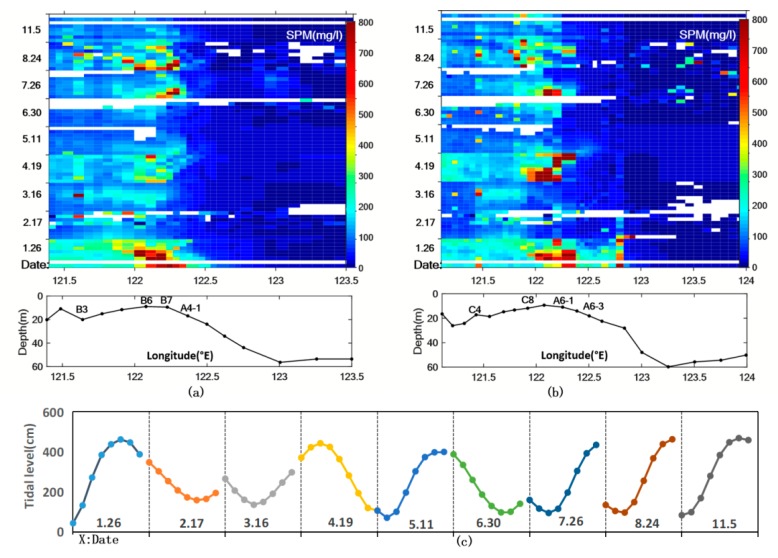
The SPM diurnal variation along two transects in the Changjiang Estuary computed by GOCI on 26 January, 17 February, 16 March, 19 April, 11 May, 30 June, 26 July, 24 August and 5 November 2016 separately. (**a**) Longitude-time plots of SPM concentration from B1 to A4-6. (**b**) longitude-time plots of SPM concentration from C1 to A6-11. (**c**) Tidal elevation measured by Luchaogang gauge at the corresponding imaging time.

Further quantitative analysis were conducted along two transects locating in the south and north channels of the Changjiang Estuary respectively (Figure 1b), as shown in Figure 8. The station C1 located near the bifurcation point of the North and South Branch of the estuary, where the influence of tide was relatively small [53], its SPM concentration revealed the fluvial regime much more. In addition, the relatively stable level of SPM concentration from C1 to C4 implied a little diurnal variation of the sediment discharged into the sea. Downstream from C4, SPM concentrations in the inner estuary exhibited similar negative correlation with tidal elevation. The higher SPM concentration in the two transects is concentrated near the estuary, presenting different magnitudes and extents covaried with tidal flow, which also approves precious observations that the tidal current plays a significant role in the SPM variation [46,47,54]. The maximum SPM concentration usually appeared during the shift of tidal flow, which may aggregate the vertical mixture. Frequent coverage of the GOCI makes it possible to catch the features of SPM distribution in surface layer much more effectively. The minimum variation occurred in the offshore waters (starting from A4-1, A6-3 separately in two transects) seemed mostly due to the relatively deeper water depth and less sediment transportation.

**Figure 9:**
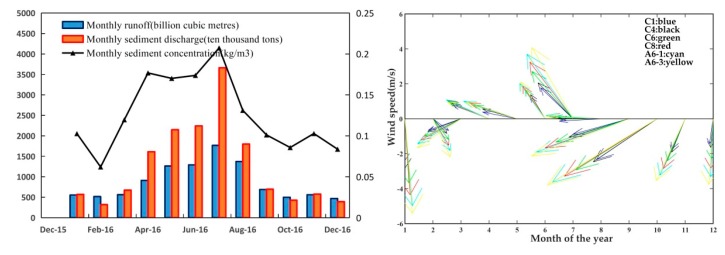
(**a**) Monthly-averaged diluted water discharge and sediment discharge in the Changjiang Estuary. (**b**) Monthly-averaged wind vectors corresponding to the representative stations (C1, C4, C6, C8, A6-3).

The influence of fluvial discharge on SPM variation is analyzed at monthly scale. The Changjiang River carries a large amount of sediments annually and discharges into the estuary and its adjacent waters, and produces various processes such as transportation, deposition and resuspension under the action of dynamics, which may be the major reason for the observed monthly and seasonal variations. Records shown in the Changjiang River sediment Bulletin in 2016 (presented in Figure 9a), the amount of SPM inputted by the Changjiang River is much higher in the wet season with large discharge from April to August, than in the dry season with low discharge from September through March. The peaking loading rate, roughly 24.22% of the annual rate occurs in the month of July. The lowest loading rate, about 0.2% of the annual rate occurs in February. However, the lower average SPM concentration in offshore waters in wet season indicates that the discharged sediment was trapped in inshore waters and cannot directly influence the distribution of SPM out of the estuary. It is more likely the mixture of the recent discharged sediments from the Changjiang River and sediments resuspended from the sea bed that had already accumulated in the estuarine delta ([55,56]).

Moreover, wind is also an important factor to affect the surface SPM distribution by enhancing vertical mixing and bring bottom sediment upwards [57]. Based on the wind vectors obtained from the remote sensing system, we can see that wind speed in offshore regions was larger than the inner part of the estuary, and the speed increased seawards (Figure 9b). The wind direction varied from the northwestern to the southwestern and then veered to the northeastern through the year. And the transport of the SPM carried by the costal current also seemed to transfer seasonally with wind direction, such as the southwards in the winter (December to February) and the northwards in the summer (June to August).

**Figure 10:**
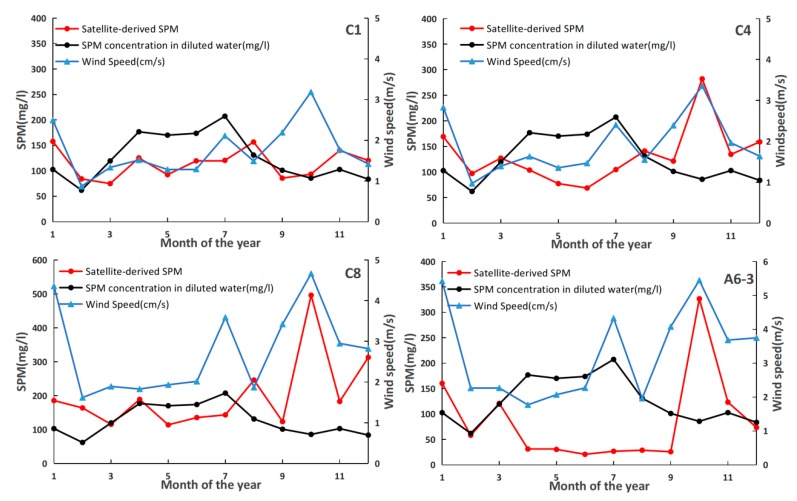
The wind speed and the Changjiang River sediments discharge at selected representative stations.

We took C1, C4, C8 and A6-3 as special samples to quantify the influences of wind and sediment discharge on SPM variation in different regions. C1 exhibited fluvial regime much more with a weak relation with wind speed (Figure 10). To the contrary, SPM in C4 and C8 covaried closely with wind speed for the correlation coefficients (r) of 75.23% and 50.98% respectively. While, the monthly averaged SPM concentration in A6-3 was generally much lower than the SPM concentration in the diluted water, which could due to the less sediment arrived with the far distance from the upstream. Without the respect of other factors, the higher SPM in the October found in A6-3 was mostly contributed by the wind induced mixing. Field surveys conducted by Bian et al. [57] showed that mixed layer depth of the East China Sea can arrive at 40 m in autumn and winter, making more turbid water mixed into the surface layer. However, the relation between wind and SPM concentration in A6-3 could be weakened by the stratification of the water column, corresponding to the weak relation during the summer.

## 5. Conclusions

In this paper, we took the Changjiang Estuary as a sampling region to prove the ability of GOCI in coastal SPM monitoring. With the results derived from GOCI images, we carried out several analyses about the influence of tidal current, wind and sediment discharge on the distribution of SPM. Our findings are summarized as follows:

(1) The atmospheric correction algorithm (UV-AC) proposed by He et al. [42] has been further validated with field data measured in the East China Sea, showing its potential in coastal turbidity waters. On the basis of the UV-AC algorithm, the GOCI level 2C data that have been corrected with Rayleigh scattering can be used for other studies in monitoring oceanic dynamics after a simpler process.
(2) The empirical model with variable of R_rs_745/R_rs_490 performed the best with whole range of SPM concentration. Comparison between the models estimated and the measured SPM shows that the modified model is of higher accuracy than other models for the application in the Changjiang Estuary. The validation results of two kinds of validation dataset, field measured and satellite-derived, indicated that the empirical algorithm calibrated in this paper can compute reliable SPM concentration for turbid coastal waters in the Changjiang Estuary. However, the algorithm gives a poor performance in offshore waters with lower SPM concentration (SPM < 35 mg/L), due to the increasing proportion of organic materials (biomass debris and phytoplankton) in suspended particle.
(3) Comparing with the daily eight images served by GOCI, we found that tide is the main driver for the diurnal variation of SPM in the Changjiang Estuary. Especially, waters near the estuary respond well with tidal elevation. Due to the diversity of phase and swing, the variation of SPM concentration during the daytime is diverse.
(4) The higher SPM concentration in dry seasons was more likely contributed by sediment resuspended from the sea bed for the less sediment discharged from the Changjiang River. Wind induced mixing also plays a key role in sediment resuspension to cause the increase of SPM concentration in surface layer.

Finally, there are still some aspects that need to be improved in the future study:

(1) It is necessary to partition SPM in future research. The weak relation between R_rs_ and SPM concentration in slightly and moderately turbid waters could only be solved by separate feature analysis on PIM and POM.
(2) The physical, geological, chemical and biological processes in the study area are so complex that we need more data to analyze the mechanisms of SPM variation spatially and temporally.

## Figures and Tables

**Table 1:** SPM retrieval models that can applied in GOCI with alternative bands. Equations are listed as they were published in the reference papers, where R_rs_(λ) is the remote sensing reflectance at the wavelength of λ nm. R^2^ is the determinate coefficient of calibrated model.

References	Band(s)	Model	SPM Range(g/m^3^)	R^2^	Study Area
Huang et al. (2015) [21]	R_rs_(745) R_rs_(680) R_rs_(555)	$\begin{array}{l} \mathrm{SPM}=2.3224/(1+\exp(-\frac{R_{\mathrm{rs}}745+R_{\mathrm{rs}}680}{R_{\mathrm{rs}}555}\\ -0.6063)/0.4211) \end{array}$ $\begin{array}{l} \mathrm{SPM}=2.2135/(1+\exp(-R_{\mathrm{rs}}745/\\ R_{\mathrm{rs}}555-0.1223/0.1439))) \end{array}$	5–300	0.81 0.83	Taihu Lake China
Chio et al. [22] (2012)	R_rs_(660)	$\mathrm{SPM}=1.7532\times \exp(204.26\times R_{\mathrm{rs}}660)$	1.88–183.1	0.9339	coastal zone adjacent to the city of Mokpo
He et al. [19] (2013)	R_rs_(745) R_rs_(490)	${\mathrm{SPM}\;=\;10}^{1.0785+1.1230\times R}{,\;R\;=\;R}_{\mathrm{rs}}{745/R}_{\mathrm{rs}}490$	8–5275	0.96	Changjiang Estuary and Hang zhou Bay
Zhongfeng Qiu [23]	R_rs_(680) R_rs_(555)	$\log(\mathrm{SPM})=1.988\times {\left(\frac{R_{\mathrm{rs}}680}{R_{\mathrm{rs}}555}\right)}^{0.877}$	1.9–1896.5	0.95	Yellow river estuary
Zhang et al. [11] Siswanto et al. [24]	R_rs_(645) R_rs_(555) R_rs_(488)	$\begin{array}{l} \log_{10}\mathrm{SPM}=0.6311+22.2158\times x_{1}-0.5239\times x_{2}\\ x_{1}=R_{\mathrm{rs}}555+R_{\mathrm{rs}}645,x_{2}=R_{\mathrm{rs}}488/R_{\mathrm{rs}}555; \end{array}$	0.66–300	/	The Yellow and East China Sea
Chen et al. [26]	R_rs_(865) R_rs_(765)	$\begin{array}{l} \mathrm{SPM}(kg/m^{3})=a\times [R_{\mathrm{rs}}{}^{-1}(\lambda_{1})-R_{\mathrm{rs}}{}^{-1}(\lambda_{2})]+b\\ \lambda_{1}=869,865;\lambda_{2}=748,765,761 \end{array}$	100–800	0.876–0.8917	Changjiang River Estuary

**Table 2:** Dates and sample numbers for the four conducted cruises and the statistics characteristics of the SPM concentration collected in the Changjiang Estuary and adjacent areas (STDEV, standard deviation).

	Max	Min	Average	STDEV
1. Calibration dataset measured in March and July, 2016 and August 2017, respectively, **48** stations				
SPM (mg/)	2061.25	10.54	209.35	353.68
Chla (mg/m^3^)	13.17	0.05	1.33	2.16
Salinity (psu)	33.85	0.14	25.29	10.14
2. Validation dataset measured in March and July, 2016 and August 2017, respectively, **12** stations				
SPM (mg/L)	812.70	10.02	180.05	251.19
Chla (mg/m^3^)	3.04	0.06	0.92	0.85
Salinity (psu)	32.61	0.71	21.37	11.88
3. Validation dataset measured in July 2018, **28** stations				
SPM (mg/L)	420.50	1.94	33.49	91.97
Chla (mg/m^3^)	34.68	0.18	4.15	6.90

**Table 3:** Comparative results between the satellite-derived R_rs_ calculated with two atmospheric correction algorithms and in-situ measured R_rs_.

Wavelength	GOCI-AC			UV-AC		
	RMSE (sr^−1^)	MARE (%)	r	RMSE (sr^−1^)	MARE (%)	r
412 nm	0.00644	29.14	0.28020	0.00612	26.85	0.21527
443 nm	0.00778	26.54	0.18683	0.00705	16.71	0.26685
490 nm	0.00944	19.68	0.19588	0.00870	14.39	0.27601
555 nm	0.01113	10.39	0.35722	0.01046	16.32	0.43746
660 nm	0.00885	27.05	0.60132	0.00767	14.23	0.68263
680 nm	0.00889	30.57	0.59925	0.00760	14.77	0.69191
745 nm	0.00561	39.17	0.09856	0.00575	32.44	0.54125

## References

[1] Gordon H.R., McCluney W.R. (1975). Estimation of the depth of sunlight penetration in the sea for remote sensing. Appl. Opt..

[2] Cloern J.E. (1987). Turbidity as a control on phytoplankton biomass and productivity in estuaries. Cont. Shelf Res..

[3] Bowers D.G., Binding C.E. (2006). The optical properties of mineral suspended particles: A review and synthesis. Estuar. Coast. Shelf Sci..

[4] Zhao H., Chen Q., Walker N.D., Zheng Q., MacIntyre H.L. (2011). A study of sediment transport in a shallow estuary using MODIS imagery and particle tracking simulation. Int. J. Remote Sens..

[5] Turner A., Millward G.E. (2002). Suspended particles: Their role in estuarine biogeochemical cycles. Estuar. Coast. Shelf Sci..

[6] Komick N.M., Costa MP F., Gower J. (2009). Bio-optical algorithm evaluation for MODIS for western Canada coastal waters: An exploratory approach using in situ reflectance. Remote Sens. Environ..

[7] Novoa S., Doxaran D., Ody A., Lafon V., Lubac B., Gernez P. (2017). Atmospheric Corrections and Multi-Conditional Algorithm for Multi-Sensor Remote Sensing of Suspended Particulate Matter in Low-to-High Turbidity Levels Coastal Waters. Remote Sens..

[8] Petus C., Marieu V., Novoa S., Chust G., Bruneau N., Froidefond J.M. (2014). Monitoring spatio-temporal variability of the Adour River turbid plume (Bay of Biscay, France) with MODIS 250-m imagery. Cont. Shelf Res..

[9] Lorthiois T., Chami M. (2012). Daily and seasonal dynamics of suspended particles in the Rhône River plume based on remote sensing and field optical measurements. Geo-Mar. Lett..

[10] Chen S., Han L., Chen X., Li D., Sun L., Li Y. (2015). Estimating wide range Total Suspended Solids concentrations from MODIS 250-m imageries: An improved method. ISPRS J. Photogramm. Remote Sens..

[11] Zhang M.W., Tang J.W., Dong Q., Song Q., Ding J. (2010). Retrieval of total suspended matter concentration in the Yellow and East China Seas from MODIS imagery. Remote Sens. Environ..

[12] Lahet F., Stramski D. (2010). MODIS imagery of turbid plumes in San Diego coastal waters during rainstorm events. Remote Sens. Environ..

[13] Hopkins J., Lucas M., Dufau C., Sutton M., Stum J., Lauret O., Channelliere C. (2013). Detection and variability of the Congo River plume from satellite derived sea surface temperature, salinity, ocean colour and sea level. Remote Sens. Environ..

[14] Chen X., Han X., Feng L. (2015). Towards a practical remote-sensing model of suspended sediment concentrations in turbid waters using MERIS measurements. Int. J. Remote Sens..

[15] Raag L., Uiboupin R., Sipelgas L. Analysis of historical MERIS and MODIS data to evaluate the impact of dredging to monthly mean surface TSM concentration. Proceedings of the SPIE—The International Society for Optical Engineering.

[16] Han B., Loisel H., Vantrepotte V., Mériaux X., Bryère P., Ouillon S., Dessailly D., Xing Q., Zhu J. (2016). Development of a Semi-Analytical Algorithm for the Retrieval of Suspended Particulate Matter from Remote Sensing over Clear to Very Turbid Waters. Remote Sens..

[17] Ryu J.H., Choi J.K., Eom J., Ahn J.H. (2011). Temporal variation in Korean coastal waters using Geostationary Ocean Color Imager. J. Coast. Res..

[18] Binding C.E., Bowers D.G., Mitchelson-Jacob E.G. (2005). Estimating suspended sediment concentrations from ocean colour measurements in moderately turbid waters; the impact of variable particle scattering properties. Remote Sens. Environ..

[19] He X., Bai Y., Pan D., Huang N., Dong X., Chen J., Chen C., Cui Q. (2013). Using geostationary satellite ocean color data to map the diurnal dynamics of suspended particulate matter in coastal waters. Remote Sens. Environ..

[20] Kumar A., Equeenuddin S.M., Mishra D.R., Acharya B. C. (2016). Remote monitoring of sediment dynamics in a coastal lagoon: Long-term spatio-temporal variability of suspended sediment in Chilika. Estuar. Coast. Shelf Sci..

[21] Huang C., Yang H., Zhu A.X., Zhang M., Lü H., Huang T., Zou J., Li Y. (2015). Evaluation of the Geostationary Ocean Color Imager GOCI to monitor the dynamic characteristics of suspension sediment in Taihu Lake. Int. J. Remote Sens..

[22] Choi J.K., Park Y.J., Ahn J.H., Lim H. S., Eom J., Ryu J.H. (2012). GOCI, the world’s first geostationary ocean color observation satellite, for the monitoring of temporal variability in coastal water turbidity. J. Geophys. Res. Oceans.

[23] Qiu Z. (2013). A simple optical model to estimate suspended particulate matter in Yellow River Estuary. Opt. Express.

[24] Siswanto E., Tang J., Yamaguchi H., Ahn Y.H., Ishizaka J., Yoo S., Kim S.W., Kiyomoto Y., Yamada K., Chiang C., Kawamura H. (2011). Empirical ocean-color algorithms to retrieve chlorophyll- a, total suspended matter, and colored dissolved organic matter absorption coefficient in the Yellow and East China Seas. J. Oceanogr..

[25] Dorji P., Fearns P., Broomhall M. (2016). A Semi-Analytic Model for Estimating Total Suspended Sediment Concentration in Turbid Coastal Waters of Northern Western Australia Using MODIS-Aqua 250 m Data. Remote Sens..

[26] Chen J., D’Sa E., Cui T., Zhang X. (2013). A semi-analytical total suspended sediment retrieval model in turbid coastal waters: A case study in Changjiang River Estuary. Opt. Express.

[27] Stavn R.H., Richter S.J. (2008). Biogeo-optics: Particle optical properties and the partitioning of the spectral scattering coefficient of ocean waters. Appl. Opt..

[28] Long C.M., Pavelsky T.M. (2013). Remote sensing of suspended sediment concentration and hydrologic connectivity in a complex wetland environment. Remote Sens. Environ..

[29] Milliman J.D., Syvitski JP M. (1992). Geomorphic/Tectonic Control of Sediment Discharge to the Ocean: The Importance of Small Mountainous Rivers. J. Geol..

[30] Xu K., Milliman J.D. (2009). Seasonal variations of sediment discharge from the Yangtze River before and after impoundment of the Three Gorges Dam. Geomorphology.

[31] Yan X.H., Jo Y.H., Jiang L., Wan Z., Liu W. T., Zhan J., Du T. (2008). Impact of the Three Gorges Dam water storage on the Yangtze River outflow into the East China Sea. Geophys. Res. Lett..

[32] Wu Y., Zhang J., Liu S.M., Zhang Z.F., Yao Q.Z., Hong G.H., Cooper L. (2007). Sources and distribution of carbon within the Yangtze River system. Estuar. Coast. Shelf Sci..

[33] Xie D.F., Wang Z.B., Shu G., De Vriend H.J. (2009). Modeling the tidal channel morphodynamics in a macro-tidal embayment, Hangzhou Bay, China. Cont. Shelf Res..

[34] Mueller J.L., Davis C.O., Arnone R.A., Frouin R., Carder K.L., Lee Z.P., Steward R.G., Hooker S., Mobley C.D., McClain C.R. (2003). Above-water radiance and remote sensing measurement and analysis protocols. Ocean Optics Protocols for Satellite Ocean-Color Sensor Validation, vol. Revision 4, III: Radiometric Measurements and Data Analysis Protocols.

[35] Mobley C.D. (1999). Estimation of the remote-sensing reflectance from above-surface measurements. Appl. Opt..

[36] Strickland J.D.H., Parsons T.R. (1969). A practical handbook of seawater analysis. Fisheries Research Board of Canada. Q. Rev. Biol..

[37] Atlas R., Hoffman R.N., Ardizzone J., Leidner S.M., Jusem J.C., Smith D.K., Gombos D. (2011). A Cross-calibrated, Multiplatform Ocean Surface Wind Velocity Product for Meteorological and Oceanographic Applications. Bull. Am. Meteorol. Soc..

[38] Wang M. (1999). Validation study of the SeaWiFS oxygen A-band absorption correction: Comparing the retrieved cloud optical thicknesses from SeaWiFS measurements. Appl. Opt..

[39] Wang M. (2010). Atmospheric Correction for Remotely-Sensed Ocean-Colour Products.

[40] Wang M. (2007). Remote sensing of the ocean contributions from ultraviolet to near-infrared using the shortwave infrared bands: Simulations. Appl. Opt..

[41] Ahn J.H., Park Y.J., Ryu J.H., Lee B. (2012). Development of atmospheric correction algorithm for Geostationary Ocean Color Imager (GOCI). Ocean Sci. J..

[42] He X., Bai Y., Pan D., Tang J., Wang D. (2012). Atmospheric correction of satellite ocean color imagery using the ultraviolet wavelength for highly turbid waters. Opt. Expraess.

[43] Tzortziou M., Neale P.J., Osburn C.L., Megonigal J.P., Maie N., Jaffé R. (2008). Tidal marshes as a source of optically and chemically distinctive colored dissolved organic matter in the Chesapeake Bay. Limnol. Oceanogr..

[44] Gordon H.R. (1995). Remote sensing of ocean color: A methodology for dealing with broad spectral bands and significant out-of-band response. Appl. Opt..

[45] Shi W., Wang M. (2010). Satellite observations of the seasonal sediment plume in central East China Sea. J. Mar. Syst..

[46] Yuan D., Zhu J., Li C., Hu D. (2008). Cross-shelf circulation in the Yellow and East China Seas indicated by MODIS satellite observations. J. Mar. Syst..

[47] Dong L.X., Guan W.B., Chen Q., Li X. H., Liu X. H., Zeng X. M. (2011). Sediment transport in the Yellow Sea and East China Sea. Estuar. Coast. Shelf Sci..

[48] Luo Z., Zhu J., Wu H., Li X. (2017). Dynamics of the Sediment Plume Over the Yangtze Bank in the Yellow and East China Seas. J. Geophys. Res..

[49] Röttgers R., Heymann K., Krasemann H. (2014). Suspended matter concentrations in coastal waters: Methodological improvements to quantify individual measurement uncertainty. Estuar. Coast. Shelf Sci..

[50] Stavn R.H., Rick H.J., Falster A.V. (2009). Correcting the errors from variable sea salt retention and water of hydration in loss on ignition analysis: Implications for studies of estuarine and coastal waters. Estuar. Coast. Shelf Sci..

[51] Bowers D.G., Braithwaite K.M., Nimmosmith WA M., Graham G.W. (2009). Light scattering by particles suspended in the sea: The role of particle size and density. Cont. Shelf Res..

[52] Gordon H.R., Wang M. (1994). Retrieval of water-leaving radiance and aerosol optical thickness over the oceans with SeaWiFS: A preliminary algorithm. Appl. Opt..

[53] Chen S., Zhang G., Yang S., Yu Z. (2004). Temporal and spatial changes of suspended sediment concentration and resuspension in the Yangtze River Estuary and its adjacent waters. Acta Geogr. Sin..

[54] Rong Z., Li M. (2012). Tidal effects on the bulge region of Changjiang River plume. Estuar. Coast. Shelf Sci..

[55] Xie Q.C., Zhang L.F., Zhou F.G. (1983). Features and transportation of suspended matter over the continental shelf off the Changjiang Estuary. Proceeding of the International Symposium on the Sedimentation on the Continental Shelf, with Special Reference to the East China Sea, Hangzhou, China, 12–16 April 1983.

[56] Shen H.T., Pan D.A. (2001). Turbidity Maximum in the Changjiang Estuary.

[57] Bian C., Jiang W., Quan Q., Wang T., Greatbatch R.J., Li W. (2013). Distributions of suspended sediment concentration in the Yellow Sea and the East China Sea based on field surveys during the four seasons of 2011. J. Mar. Syst..

